# Second-line Hormonal Therapy for the Management of Metastatic Castration-resistant Prostate Cancer: a Real-World Data Study Using a Claims Database

**DOI:** 10.1038/s41598-020-61235-4

**Published:** 2020-03-06

**Authors:** Jui-Ming Liu, Cheng-Chia Lin, Kuan-Lin Liu, Cheng-Feng Lin, Bing-Yu Chen, Tien-Hsing Chen, Chi-Chin Sun, Chun-Te Wu

**Affiliations:** 1 Division of Urology, Department of Surgery, Taoyuan General Hospital, Ministry of Health and Welfare, Taoyuan, Taiwan; 2 Graduate Institute of Life Sciences, National Defense Medical Center, Taipei, Taiwan; 3 Department of Urology, Chang Gung Memorial Hospital, Keelung, Taiwan; 4 Department of Medical Research and Development, Chang Gung Memorial Hospital, Keelung, Taiwan; 5 Division of Cardiology, Department of Internal Medicine, Chang Gung Memorial Hospital, Keelung, Taiwan; 6 College of Medicine, Chang Gung University, Taoyuan, Taiwan; 7 Biostatistical Consultation Center of Chang Gung Memorial Hospital, Keelung, Taiwan; 8 Department of Ophthalmology, Chang Gung Memorial Hospital, Keelung, Taiwan; 9 Department of Chinese Medicine, College of Medicine, Chang Gung University, Taoyuan, Taiwan

**Keywords:** Cancer, Prostate

## Abstract

We evaluated the efficacy of second-line hormonal therapy for treatment of metastatic castration-resistant prostate cancer (mCRPC) in a real-world retrospective study. We conducted a population-based real-world cohort study of 258 mCRPC patients between 2014 and 2018 using the Chang Gung Research Database (CGRD) of Taiwan. The second-line hormonal therapy included abiraterone acetate and enzalutamide. The clinical efficacy outcomes were overall survival (OS) and prostate-specific antigen (PSA) doubling time. The median PSA level was also assessed. In total, 223 mCRPC patients who underwent second-line hormonal therapy met all of the inclusion and exclusion criteria for this study. Among them, 65 (29.1%) patients were in the PSA response group and 158 (70.9%) were in the non-response group. The median age was 72.9 years. The median OS was 12.3 months (range: 9.9–19.9 months) and 9.6 months (range: 5.3–15.9 months) in the response and non-response groups, respectively, and the respective PSA doubling times were 9.0 months (range: 4.4–11.6 months) and 3.9 months (range: 2.2–9.1 months), with a median follow-up period of 10.5 months. A significantly longer median OS was seen in the PSA response group. This real-world database study demonstrated that clinical outcomes of second-line hormonal therapy were better in patients with a PSA response. Further studies are warranted to achieve a better understanding of second-line hormonal therapy for mCRPC in Asian populations.

## Introduction

Androgen deprivation therapy (ADT) has been a mainstay of treatment for advanced prostate cancer (PCa)^1^. Eventually, patients with PCa will progress to castration-resistant prostate cancer (CRPC). In recent years, second-line hormonal therapies for metastatic CRPC (mCRPC) have become available, including abiraterone acetate (AA) and enzalutamide^2,3^. Both of them prolonged overall survival in patients with mCRPC who had disease progression.

A randomized double-blind controlled study (COU-AA-301) of metastatic CRPC patients post-chemotherapy showed that AA therapy significantly increased overall survival (OS) and radiographic progression-free survival (rPFS) in comparison to placebo^2^. The COU-AA-302 trial also showed that AA increased OS and rPFS in metastatic CRPC patients pre-chemotherapy^4^. Meanwhile, the AFFIRM and PREVAIL studies showed that enzalutamide had significant benefits on OS and rPFS in metastatic CRPC patients, both post- and pre-chemotherapy^3,5^. AA and enzalutamide for treatment of metastatic CRPC have been approved by the Taiwan Food and Drug Administration since August 2013 and 2015, respectively^6^. Several clinical trials have been performed in Taiwan^7,8^.

Information on time to treatment failure and factors responsible for response versus non-response to treatment in a real-world setting is important to support the findings of randomized controlled trials of Asian populations. To date, no comprehensive analysis of the relationship between second-line hormonal therapy and the clinical outcomes of mCRPC in Asian patients has been performed. Therefore, the aim of this study is to investigate the clinical benefit of second-line hormonal therapy in Asian patients using real-world data from the Chang Gung Research Database (CGRD).

## Methods

### Data source and collection

The Chang Gung Medical Foundation (CGMF), which consists of seven Chang Gung Memorial Hospitals (CGMHs), is the largest medical system in Taiwan. CGMF has 10,070 beds, with admission of more than 280,000 patients each year^9^. All seven CGMHs use electronic medical records (EMRs) for medical practice. The CGRD is a deidentified database comprised of multi-institutional standardized EMRs, dating back to 2000, from seven CGMHs in northern and southern Taiwan: two medical centers, two regional hospitals, and three district hospitals^10^.

The CGRD contains clinical epidemiological data, as well as laboratory test data, inpatient and outpatient data, emergency service use data, pathological reports, nursing data, disease category data, surgery data, and cancer registry data^10^. The clinical diagnoses of database patients were determined according to the International Classification of Diseases, 9th revision, Clinical Modification (ICD-9-CM) and ICD-10-CM codes. This study was approved by the Institutional Review Board of Chang Gung Memorial Hospital (approval number: CGMHIRB No. 201900278B0) As this was a retrospective study and all data was anonymous, the Institutional Review Board department agreed with the authors that it waived the need for informed consent. All methods were performed in accordance with the relevant guidelines and regulations of Institutional Review Board of Chang Gung Memorial Hospital.

### Study population

The study subjects were selected based on the CGRD data for the period January 1^st^, 2014 to December 31^st^, 2017. The accuracy of the PCa diagnoses of the subjects was confirmed by ICD-9-CM or ICD-10-CM codes plus combined with definitive pathology reports. The end of follow-up period was on June 30^th^, 2018.

**Figure 1:**
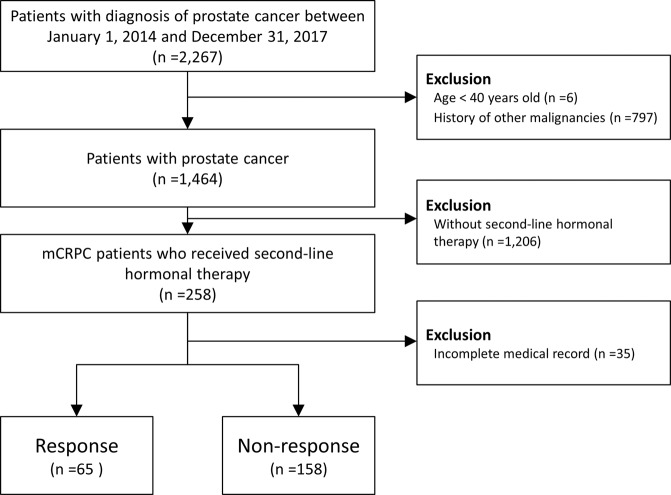
Patient selection.

All patients with PCa who had follow-up data for at least 180 days after the initial PCa diagnosis were enrolled in the study (Fig. 1). In general, patients who were newly diagnosed with PCa (ICD-9-CM: 185 and ICD-10-CM: C61)^11^ between 2014 and 2017 were included in this study. The mCRPC patients who received second-line hormonal therapy were also considered for inclusion. In Taiwan, second-line hormonal therapy were only reimbursement by the National Health Insurance administration for indication of mCRPC patients after confirmation by experts assigned. The exclusion criteria were age younger than 40 years at the time of diagnosis^12^, previous history of any malignancy, and incomplete medical records.

The enrolled patients were then divided into response and non-response groups according to their prostate-specific antigen (PSA) response. PSA response and clinical, biochemical progressive disease were defined according to the Prostate Cancer Clinical Trials Working Group (PCWG3) criteria^13^. A decline in PSA of 50%, confirmed by a second evaluation at 3 weeks after the first, was considered to indicate a PSA response in the absence of any evidence of disease progression in imaging studies.

### Study outcomes and covariates

Patients in the CGRD who underwent second-line hormonal therapy were identified. Second-line hormonal therapies include AA and enzalutamide. Other second-line hormonal medications, such as apalutamide and darolutamide, are not covered by the National Health Insurance system of Taiwan, so no such patients were enrolled in this study.

The primary outcome was OS, defined as the time between the first dose of second-line hormonal therapy and death. The PSA doubling time and PSA level (ng/mL) were also assessed in this study. Censoring was defined as death or until the end of the follow-up period on June 30^th^, 2018 (whichever came first).

The patients were classified into the following five age groups: <50 years, 50–60 years, 60–70 years, 70–80 years, and ≥ 80 years. The following comorbidities of mCRPC were included in the analysis as covariates: diabetes mellitus, hypertension, hyperlipidemia, coronary heart disease, cerebrovascular disease, chronic obstructive pulmonary disease(COPD), chronic kidney disease, chronic liver disease, and the Charlson comorbidity index (CCI)scores^14^. Data on initial stage of PCa and Gleason scores were also included in the analysis. Baseline laboratory data included hemoglobin, blood urea nitrogen (BUN), creatinine (Cr), aspartate aminotransferase (AST), alanine aminotransferase (ALT), lactate dehydrogenase (LDH), sodium (Na), and potassium (K) levels, and white blood cell (WBC) and platelet counts.

### Statistical analysis

The baseline characteristics of the patients were first analyzed using descriptive statistics. Cox proportional hazards models were used to compare OS and PSA doubling time between the response and non-response groups. Kaplan-Meier curve analysis was used to estimate the cumulative OS for the two groups, and the group difference therein was assessed using the log-rank test. The covariates were also subjected to multivariate analyses, i.e., Cox proportional hazards regression models including hazard ratios (HRs) and 95% confidence intervals (CIs). Analyses were conducted using SAS software (version 9.4; SAS Institute Inc., Cary, NC, USA).

### Ethical standards

Ethics approval was obtained from the Institutional Review Board of Chang Gung Memorial Hospital (approval number: CGMHIRB No. 201900278B0). As all data were anonymized from existing databases and results were presented in aggregate; the requirement for informed consent was waived.

## Results

A total of 223 mCRPC patients who underwent second-line hormonal therapy and met all of the inclusion criteria were enrolled in this study. The median age was 72.9 years (interquartile range (IQR): 65.8–78.5 years), and the median follow-up period was 10.5 months (IQR: 6.4–17.4 months). The demographic characteristics of the patients are shown in Table 1. In total, 65 (29.1%) patients showed a PSA decline of more than 50% from baseline and were included in the response group, and 158 (70.9%) were included in the non-response group. Compared to the non-response group, the response group was slightly younger and had a higher prevalence of diabetes. The response group had similar Gleason scores and clinical stages to the non-response group. There was no difference in baseline laboratory data, except for a higher LDH in the non-response group (Table 1).

**Table 1:** Demographic characteristics of study subjects.

Variable		All	Response	Non-response	*P*-value
		(n = 223)	(n = 65)	(n = 158)	
Age, median (IQR)		72.9 (65.8–78.5)	72.7 (65.9–75.8)	73.1 (65.8–79.0)	0.46
Age group (%)	<50	1 (0.4%)	0 (0.0%)	1 (0.6%)	0.88
	50–60	19 (8.5%)	5 (7.7%)	14 (8.9%)	
	60–70	67 (30.0%)	20 (30.8%)	47 (29.7%)	
	70–80	97 (43.5%)	31 (48.1%)	66 (41.8%)	
	≥80	39 (17.5%)	9 (13.5%)	30 (19.0%)	
Comorbidity (%)	Diabetes	73 (32.7%)	31 (48.1%)	42 (26.6%)	<0.01*
	Hypertension	123 (55.2%)	39 (59.6%)	84 (53.2%)	0.42
	Hyperlipidemia	53 (23.8%)	20 (30.8%)	33 (20.9%)	0.14
	Coronary heart disease	38 (17.0%)	8 (11.5%)	30 (19.0%)	0.22
	Cerebrovascular disease	32 (14.3%)	11 (17.3%)	21 (13.3%)	0.47
	COPD	39 (17.5%)	11 (17.3%)	28 (17.7%)	0.95
	Chronic kidney disease	63 (28.3%)	23 (34.6%)	40 (25.3%)	0.19
	Chronic liver disease	63 (28.3%)	15 (23.1%)	48 (30.4%)	0.31
CCI score, median (IQR)		4 (3–5)	4 (3–5)	4 (3–5)	0.74
BMI, median (IQR)		24.3 (22.5–26.4)	24.2 (22.3–26.3)	24.3 (22.8–26.6)	0.97
BMI (%)	<30	100 (44.8%)	29 (44.3%)	71 (44.9%)	0.63
	>=30	7 (3.1%)	3 (3.8%)	4 (2.6%)	
	Unknown	116 (52.1%)	33 (51.9%)	83 (52.5%)	
Stage (initial)	I	3 (1.3%)	0 (0.0%)	3 (1.9%)	0.63
	II	14 (6.3%)	5 (7.7%)	9 (5.7%)	
	III	11 (4.9%)	5 (7.7%)	6 (3.8%)	
	IV	168 (75.3%)	50 (76.9%)	118 (74.7%)	
	Unknown	27 (12.2%)	5 (7.7%)	22 (13.9%)	
Gleason score, median (IQR)		9 (8–9)	9 (8–9)	9 (8–9)	0.88
Gleason score (%)	<=7	19 (8.5%)	8 (11.5%)	11 (7.0%)	0.63
	8	21 (9.4%)	6 (9.6%)	15 (9.5%)	
	>=9	88 (39.5%)	25 (38.5%)	63 (39.9%)	
	Unknown	95 (42.6%)	26 (40.4%)	69 (43.7%)	
Lab data	Hb (g/dL)	11.0 (9.3–12.7)	11.0 (9.1–12.3)	11.1 (9.3–12.7)	0.75
	BUN (mg/dL)	18.1 (13.8–22.4)	18.3 (13.2–21)	18.1 (13.8–22.8)	0.97
	Cr (mg/dL)	0.9 (0.8–1.2)	1.0 (0.8–1.2)	0.9 (0.8–1.2)	0.59
	AST (U/L)	24.0 (21.0–33.0)	24.0 (20.0–35.0)	24 (21.0–33.0)	0.98
	ALT (U/L)	17.0 (14.0–25.0)	19.0 (15.0–27.0)	17 (14.0–25.0)	0.43
	LDH (U/L)	230.0 (185.0–276.0)	209.0 (175.0–254.0)	235.2 (191.0–295.0)	0.04*
	Na (mEq/L)	139.0 (136.0–141.0)	139.0 (136.0–140.0)	139.0 (136.0–141.0)	0.29
	K (mEq/L)	4.1 (3.8–4.3)	4.1 (3.8–4.5)	4.1 (3.7–4.3)	0.38
	WBC (1000/μL)	7.3 (5.8–8.7)	7.3 (6.7–9.9)	7.3 (5.3–8.7)	0.17
	Platelet (1000/μL)	212.5 (167.0–226.0)	221 (167.0–224.0)	209.0 (179.0–226.0)	0.34

*p < 0.05.

Abbreviations: COPD, chronic obstructive pulmonary disease; IQR, interquartile range; BMI, body mass index; CCI, Charlson Comorbidity Index; Hb, hemoglobin; BUN, blood urea nitrogen; Cr, creatinine; AST, aspartate aminotransferase; ALT, alanine aminotransferase; LDH, lactate dehydrogenase; Na, sodium; K, potassium; WBC, white blood cell.

**Figure 2:**
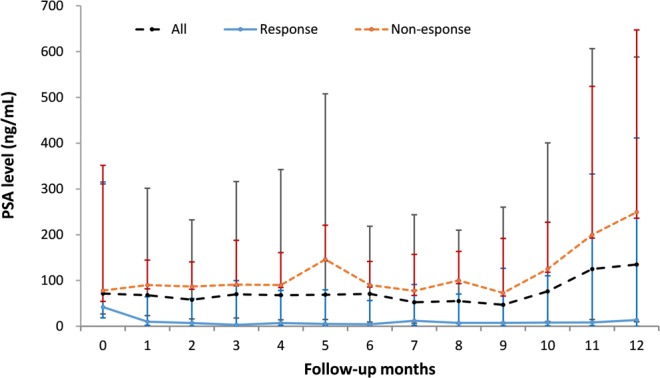
The median PSA levels in response and non-response groups of mCRPC patients with second-line hormonal therapy. mCRPC: metastatic castration-resistant prostate cancer.

The median PSA levels are shown in Fig. 2. The response group had a significantly lower PSA level during the entire study period. The median initial PSA level was 402.6 ng/mL in the response group and 554.4 ng/mL in the non-response group. The PSA level was rapidly lower to nadir level after 1 month of second-line hormonal therapy in the response group. The mean PSA levels of the study subjects are shown in Supplementary Fig. S1.

**Figure 3:**
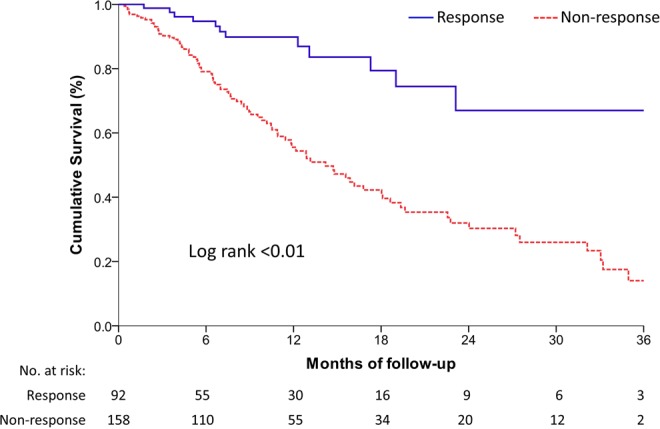
The cumulative survival calculated by the Kaplan-Meier analysis of mCRPC patients with second-line hormonal therapy. mCRPC: metastatic castration-resistant prostate cancer.

The median OS was 12.3 months (range: 9.9–19.9 months) and 9.6 months (range: 5.3–15.9 months) in the response and non-response groups, respectively (Table 2), and the respective PSA doubling times were 9.0 months (range: 4.4–11.6 months) and 3.9 months (range: 2.2–9.1 months) (Table 2). The cumulative survival calculated by the Kaplan-Meier survival curve is shown in Fig. 3. The response group had significantly better OS than the non-response group (HR: 0.34, 95% CI: 0.17–0.65, *P* < 0.01 on log-rank test).

**Table 2:** The outcome of overall survival, PSA doubling time, and final PSA level in response and non-response groups of second-line hormonal therapy.

	All	Response	Non-response	P value
	(n = 223)	(n = 65)	(n = 158)	
Overall survival (month)	10.5 (6.4–17.4)	12.3 (9.9–19.9)	9.6 (5.3–15.9)	<0.01*
PSA doubling time (month)	5.0 (2.5–9.8)	9.0 (4.4–11.6)	3.9 (2.2–9.1)	<0.01*
Final PSA level (ng/mL)	186.8 (30.5–968.1)	23.3 (0.9–187.4)	277.8 (50.1–1281.2)	0.03*

*p < 0.05.

PSA, prostatic specific antigen.

The results of multivariate analysis of factors associated with OS and PSA doubling time are shown in Supplementary Table S1. Age was a significant risk factor for OS. The outcomes of different second-line hormonal medications are listed in Supplementary Table S2. The numbers of chemo-naïve and post-chemotherapy patients are listed in Supplementary Table S3.

## Discussion

This real-world study supports the hypothesis that second-line hormonal therapy is beneficial for Asian patients with mCRPC. We enrolled a retrospective data-based cohort of 223 patients who received second-line hormonal therapy for mCRPC. We demonstrated that PSA-response group had longer OS,PSA doubling time and lower final PSA level compared to non-response group. The median PSA level of the response group decreased rapidly after the first month of therapy and then reached a nadir. Age of the mCRPC patients was significantly associated with OS.

In the current cohort study, the median OS was 12.3 and 9.6 months for the response and non-response groups, respectively. These times were much shorter than those reported for the COU-AA-301 study (15.8 months), COU-AA-302 study (34.7 months), AFFIRM study (18.4 months), and PREVAIL study (32.4 months)^2–5^. In previous studies including Asian patients, Poon *et al*. reported that the median OS with use of AA for treating mCRPC was 18.1 and 15.5 months for chemo-naïve and post-chemotherapy groups, respectively, in Hong Kong^15^. Fan *et al*. reported a median OS with use of AA for treating chemotherapy-naive mCRPC patients of 23.3 months^16^. A study including Taiwanese and Korean patients reported a median OS of 11.8 months among 82 mCPRC patients with use of AA use after chemotherapy^17^. In the JPN-201 study, the 6-month survival rate was estimated to be 81% among patients receiving AA for mCRPC post-chemotherapy, compared to 98% in chemotherapy-naive mCRPC patients^18,19^.

In post-chemotherapy mCPRC patients, the median OS with enzalutamide treatment was 10.3 months in a study performed in Japan and 15.8 months in a study done in Hong Kong^20,21^. A Korean study reported a median OS of 17.7 months with enzalutamide treatment for chemotherapy-naive mCRPC patients^22^. In our study, however, the median OS with use of AA was 9.5 months in chemotherapy-naive mCRPC patients, and 10.5 months in post-chemotherapy mCRPC patients. The median OS with enzalutamide treatment was 9.4 months in chemotherapy-naive mCRPC patients, versus 11.6 in post-chemotherapy mCRPC patients. OS was shorter than in studies performed in other Asian countries as above mentioned. We supposed that selection bias due to NHI policies in Taiwan is responsible for this difference. The AA and enzalutamide approval dates, for coverage by the NHI system of Taiwan, differed; AA was approved for use in mCPRC patients after chemotherapy in December 2014, and in chemo-naïve patients in September 2017. Meanwhile, enzalutamide was approved for use in mCPRC patients after chemotherapy in September 2016, and in chemo-naïve patients in September 2017. The AA and enzalutamide usage sequence has a substantial influence on the enrolled number of study subjects, which may have affected the outcomes (Supplementary Table S3). The patients recruited in our study had many prognostic factors result in poor prognosis. Approximately 85.1% of the patients enrolled had a Gleason score ≥8 at the time of diagnosis, in contrast to 51% in the COU-AA-301 study. We postulate that the inferior survival outcome of our study could be attributable to a relatively high tumor burden in our study cohort.

In this study, the median PSA level of response group for second-line hormonal therapy rapidly decreased from 1st month and then reach to PSA nadir at 3rd month which is compatible to the results of a Japanese study^23^. Nakayama *et al*. conducted two Japanese trials, JPN-201 and JPN-202, which included 94 mCRPC patients with AA. PSA declined from week 4, and the mean time to PSA nadir was 5.3 months in the chemotherapy-naïve mCRPC patients and 2.0 months in the post-chemotherapy patients^23^. Physician could recognize response group and non-response patient in first month after receiving 2nd line hormone therapy. It is critical for patient and physician to decide to maintain 2nd line hormone therapy. or not.

Multivariate analysis demonstrated that age was significantly associated with shorter OS in the mCRPC patients in this study. This is intuitive because age has been shown to be associated with adverse effects of second-line hormonal therapy^20^. In a Korean study, age showed an increased trend for worse OS^22^.

The strengths of this study was that it used a single large database, the hospitals of the total volume of Taiwan Chang Gung Memorial Hospital system which were more 10,000 beds and 9 branchs. In addition, the CGRD contains more than 90 sub datasets and numbers of sub datasets are still increasing. We could explore association in lab examination, pathology severity, even body weights, smoking that traditional insurance database could not performed. However, there were also several limitations to this study. The major limitation was the time lag in NHI approval between AA and enzalutamide, which may have influenced the number of subjects eligible for enrolment, leading to selection bias. In addition, there was a selection bias toward more severe patients because the CGMHs are the largest referral hospitals in Taiwan, and have larger proportions of such patients. Third, the data of patients visiting other hospitals were not available in the CGRD, such that the dataset was not complete. Fourth, data from the CGRD are not representative of the disease status of the overall Taiwanese population. Fifth, a longer follow-up period for observation of the outcomes of second-line hormonal therapies for mCRPC is warranted. Compared to non-responders, few good PSA responders may exist potential bias in our study. PSA response since a discordant response may exist between radiological and biochemical response. Due to most of the mCPRC patients were received abiraterone in this study, it is difficult to extrapolate useful data for enzalutamide. Finally, this population-based study was retrospective, and prospective studies are needed to fully evaluate the utility of second-line hormonal therapies for Asian mCRPC patient populations.

In conclusion, we conducted a real-world database study of second-line hormonal therapy for Taiwanese patients with mCRPC. The efficacy of such therapy was better in those with a PSA response. Further studies are warranted to obtain a better understanding of the outcomes of second-line hormonal therapy for mCRPC in Asian populations.

## Supplementary information

Supplementary File.
