# Supplementary material for: Second-line Hormonal Therapy for the Management of Metastatic Castration-resistant Prostate Cancer: a Real-World Data Study Using a Claims Database
Source: Sci Rep. 2020 Mar 6;10:4240. doi: 10.1038/s41598-020-61235-4 (PMC7060246; doi:10.1038/s41598-020-61235-4)

Supplementary File

Second-Line Hormonal Therapy for the Management of Metastatic Castration-Resistant Prostate Cancer: a Real-World Data Study Using a Claims Database

Jui-Ming Liu,MD; Cheng-Chia Lin,MD; Kuan-Lin Liu,MD; Cheng-Feng Lin,MD ;Bing-Yu Chen,PhD; Tien-Hsing Chen,MD; Chi-Chin Sun, MD, PhD; Chun-Te Wu,MD,PhD

We provided the supplementary file for additional information about this study

Table of Contents

Cover…..1
Table of Contents..2
Supplementary Table & Figure 3
1. Table 1 3
2. Table 2 4
3. Table 3 5
4. Figure 1 6

Supplementary Table 1. Multivariable analysis of overall survival and PSA doubling time in mCPRC patients

	Overall survival		PSA doubling	
	HR (95% CI)	P value	HR(95% CI)	P value
Age	1.03 (1.00-1.06)	0.04*	0.99 (0.96-1.02)	0.37
Stage <IV	ref		ref	
Stage IV	1.75 (0.73-4.24)	0.21	1.98 (0.93-4.21)	0.08
Diabetes	1.01 (0.59-1.74)	0.97	1.12 (0.71-1.77)	0.64
Hypertension	1.27 (0.76-2.12)	0.36	1.40 (0.86-2.28)	0.18
Hyperlipidemia	0.81 (0.44-1.50)	0.50	0.67 (0.37-1.21)	0.18
Coronary heart disease	0.67 (0.35-1.29)	0.23	1.44 (0.81-2.55)	0.21
Cerebrovascular disease	1.47 (0.73-2.98)	0.28	1.43 (0.75-2.76)	0.28
COPD	1.45 (0.82-2.54)	0.20	0.94 (0.53-1.69)	0.84
Chronic kidney disease	1.16 (0.70-1.91)	0.57	0.96 (0.60-1.53)	0.87
Chronic liver disease	1.23 (0.75-2.00)	0.41	1.23 (0.77-1.97)	0.39

*p<0.05

PSA, prostatic specific antigen ; mCPRC, metastatic castration-resistant prostate cancer; HR, hazard ratio; CI, confidence interval; COPD, chronic obstructive pulmonary disease

Supplementary Table 2. The outcome of second-line hormonal medication by types in chemo-naïve and post-chemotherapy mCRPC patients

	Abiraterone acetate		Enzalutamide	
	Chemo-naïve (n=31)	post-Chemo (n=149)	Chemo-naïve (n=22)	post-Chemo (n=21)
	median (IQR)	median (IQR)	median (IQR)	median (IQR)
PSA Response (%)	8 (25.8%)	37 (24.8%)	10 (45.4%)	10 (47.6%)
Overall survival (month)	9.5 (7.0-10.5)	10.5 (4.3-19.5)	9.4 (5.6-10.6)	11.6 (5.1-14.5)
PSA doubling time (month)	7.6 (3.3-9.1)	3.8 (2.0-9.7)	8.3 (4.5-9.7)	9.2 (4.0-12.5)
Final PSA level (ng/mL)	64.3 (5.2-372.6)	338.1 (50.1-1376.2)	23.0 (1.9-74.8)	171.6 (10.2-711.7)

Chemo, chemotherapy; mCPRC, metastatic castration-resistant prostate cancer ;IQR, interquartile range; PSA, prostatic specific antigen

Supplementary Table 3. The number of study subjects of chemo-naïve and post-chemotherapy mCRPC patients

	Abiraterone acetate		Enzalutamide	
	Chemo-naïve	post-Chemo	Chemo-naïve	post-Chemo
Year	n	n	n	n
2014	0	11	0	0
2015	2	61	0	0
2016	11	41	0	2
2017	18	36	22	19

Chemo, chemotherapy

Supplementary Figure 1. The mean PSA levels in mCRPC patients with second-line hormonal therapy.

mCRPC: metastatic castration-resistant prostate cancer

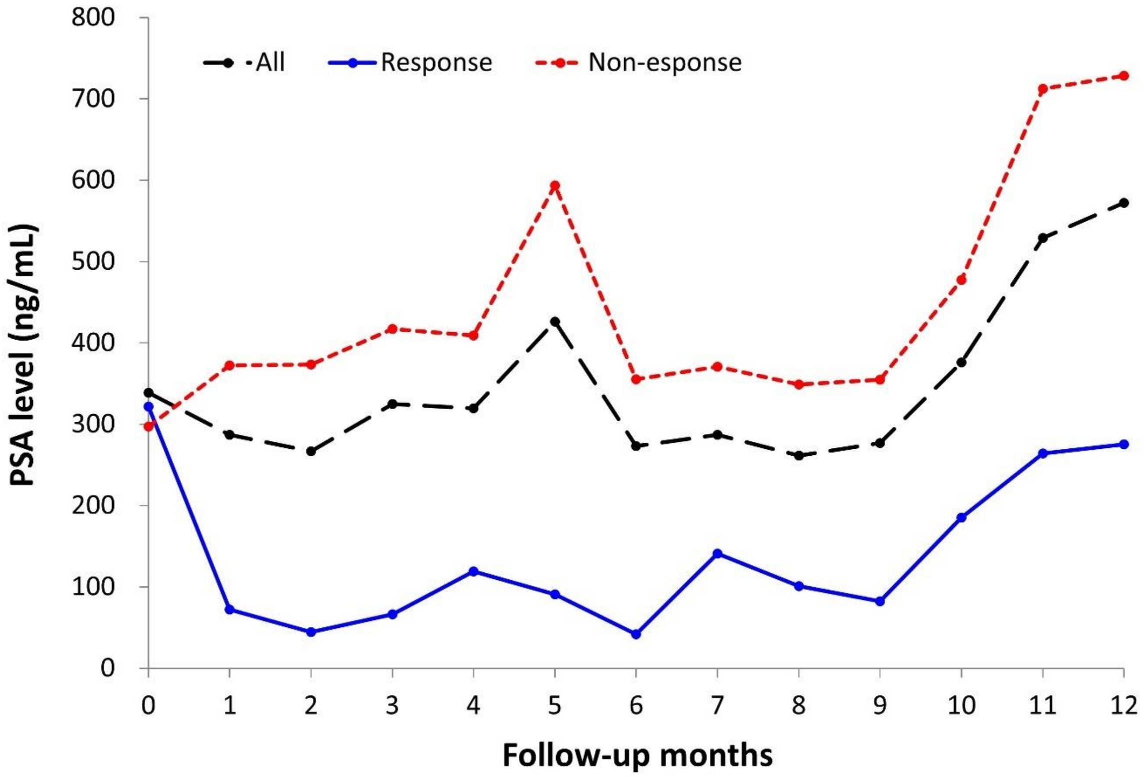

Supplement: Supplementary file 1 — Supplementary File. [file 41598_2020_61235_MOESM1_ESM.pdf]
